# Mental Health Apps Available in App Stores for Indian Users: Protocol for a Systematic Review

**DOI:** 10.2196/71071

**Published:** 2025-04-16

**Authors:** Seema Mehrotra, Ravikesh Tripathi, Pramita Sengupta, Abhishek Karishiddimath, Angelina Francis, Pratiksha Sharma, Paulomi Sudhir, Srikanth TK, Girish N Rao, Rajesh Sagar

**Affiliations:** 1 Department of Clinical Psychology National Institute of Mental Health and Neurosciences Bengaluru India; 2 E-Health Research Centre International Institute of Information Technology Bangalore Bengaluru India; 3 Department of Epidemiology, Centre for Public Health National Institute of Mental Health and Neurosciences Bengaluru India; 4 Department of Psychiatry All India Institute of Medical Sciences New Delhi India

**Keywords:** mental health apps, mHealth, review of apps, smartphone apps, MHApps for Indian users, India, mobile phones

## Abstract

**Background:**

There has been a surge in mental health apps over the past few years. While these have great potential to address the unmet mental health needs of the population, the recent proliferation of mental health apps in the commercial marketplace has raised several concerns, such as privacy, evidence-based, and quality. Although there is mounting research on the effectiveness of mental health apps, the majority of these are not accessible to the public and most of those available have not been researched. Despite the rapid growth of the digital health market in India, there are no comprehensive reviews of publicly available mental health apps for Indian users. Hence it becomes important to review mental health apps freely available to potential end users in terms of their scope, functions, and quality.

**Objective:**

This study aims to systematically evaluate mental health apps available to Indian users in app stores.

**Methods:**

This systematic review of mental health apps will be performed following the Target user, Evaluation focus, Connectedness and Health domain approach and the PASSR (Protocol for App Store Systematic Reviews) checklist. Fifteen key search terms covering various mental health conditions and therapies will be used on the Android and iOS stores. The identified apps will be further screened and reviewed based on the inclusion and exclusion criteria. The pool of eligible apps will be downloaded for detailed review. The following steps will be adopted to streamline the review process and interrater consistency. Six apps will be randomly selected from the downloaded apps, for joint discussion and review by a team of 4 primary reviewers and 2 mentors. Following this, a new set of 6 randomly selected apps will be rated independently by the primary reviewers and the differences in ratings will be jointly discussed for generating consensus. Subsequently, the primary reviewers will individually review the remaining apps in the list. Data will be extracted based on predecided parameters such as privacy policy, basic purpose, type of developer, nature of intervention strategies, and guided versus unguided nature. Additionally, the apps will be reviewed for quality using the Mobile Application Rating Scale. The data analysis and synthesis strategy will incorporate descriptive statistics based on quality evaluation using the Mobile Application Rating Scale and examining the content of the apps for generating descriptive information.

**Results:**

The initial screening of mental health apps available for Indian users on the Google Play Store and Apple App Store was initiated in October 2024. We expect to complete the detailed systematic review by April 2025.

**Conclusions:**

This study will offer a comprehensive review of mental health apps available in digital marketplaces for Indian users and has implications for end users, policy makers, developers, and mental health professionals.

**Trial Registration:**

International Platform of Registered Systematic Review and Meta-analysis Protocols INPLASY2024100035; https://inplasy.com/inplasy-2024-10-0035/

**International Registered Report Identifier (IRRID):**

DERR1-10.2196/71071

## Introduction

### Background

According to the National Mental Health Survey (2017-2018), approximately 10% of Indian adults experience a diagnosable mental health condition, and the treatment gap for most such conditions exceeds 60% [1]. For example, the treatment gap for depression in India is 85.2%. Factors contributing to this gap include the stigma surrounding mental health issues and their treatment, the uneven distribution of mental health services, affordability challenges, and the scarcity of trained mental health professionals [2]. Some of these same factors have also become the drivers of the growth of digital mental health solutions in India.

There has been an increasing popularization of telehealth and digital mental health services in India [3,4]. This has been fueled by several factors such as the rapid proliferation of smartphone users, increasing internet penetration, the emergent needs and familiarity with digital services during the COVID pandemic, government initiatives promoting digital literacy, digital health, and specifically telemental health, rising number of startups in the mental health space apart from improved awareness about mental health and felt needs for convenient and accessible solutions [4-8].

India’s smartphone penetration rate was 71% in 2023 [9]. Mental health apps have seen a 30% increase in downloads, with over 200,000 new users in the year 2023 alone. India’s digital mental health market is projected to grow at a compound annual growth rate of 28.16% and is expected to reach a projected revenue of US $62.86 million by 2032 [10].

The last few years have seen a mushrooming of mental health apps, several of which have been developed with peripheral involvement or lack of involvement of mental health professionals, and this can raise significant concerns about the validity and appropriateness of suggestions, messages, and recommendations contained in the apps [11]. Inadequately customized recommendations can delay seeking professional help and extend the period of reliance on self-help alone even when the nature and severity of the problem warrants professional help.

Globally, the last decade has also seen a plethora of scoping reviews, systematic reviews, and meta-analyses of effectiveness and efficacy studies, including a few from India on mental health apps. 1009 psychosocial wellness and stress management apps with varied content were reviewed by Lau et al [12]. Almost all were designed as purely self-help apps, with less than 2% meant to serve as a supplement to in-person therapy or involving therapy via a web-based platform. It was noted that only 4.66% of apps targeted individuals with psychiatric disorders. This review also highlighted that only 2% of the apps in app stores were supported by original research publications. Another review aimed to document the proportion of mental health apps offering comprehensive therapeutic treatments for anxiety and depression available in the app stores and developed using evidence-based frameworks. The authors found that out of the 293 apps shortlisted as offering a therapeutic treatment for anxiety and/or depression, 55.3% mentioned an evidence-based framework in their app store descriptions. However, only 6.2% had published evidence of their efficacy [13].

Alqahtani and Orji [14] examined more than 13,000 user reviews of 106 publicly available mental health apps in app stores. Their review highlighted the value placed by end users on the user interface, user-friendliness, and adaptive functionalities. Lack of variety of content, low scope for personalization, lack of customer service, trust, and privacy issues were cited as some of the pitfalls [14]. In another review, 104 mental health apps on Google Play and App Store were examined through sentiment analysis of 88,125 user reviews, using machine learning and thematic analysis. The emergent negative themes spanned usability, content, ethics, customer support, and billing issues. The positive themes included appealing interface, app stability, customizability, high-quality and diversity of content, personalization, privacy and security, and low subscription cost [15].

Some of the recent research papers on mental health apps continue to use App Store star ratings as a proxy for quality and satisfaction, although there are growing concerns with this metric. Despite the potential clinical applications and benefits, the app marketplace, with an estimated 3,00,000 health apps and 10,000 focused on mental health, poses enormous challenges to users who wish to explore and find apps that may suit their needs [16]. Insufficient regulation of health apps in the commercial marketplace can result in several apps making unsubstantiated claims, offering inaccurate and potentially dangerous information, or posing threats to user privacy. Recent research has shown that the measures that are currently used in the app marketplace for the purpose of evaluating apps (eg, app downloads or star ratings) do not provide adequate representation of apps in terms of important metrics such as security, engagement, or effectiveness [17,18].

Studies on mental health apps and reviews from India have been scarce. An overview of smartphone apps aimed at suicide prevention available for Indian users on Google Play Store found that only 11.62% of 43 apps reviewed provided information about a formal evaluation process or study [19]. It was also noted that only about 16% of the apps intended for direct use by people at suicidal risk had an initial screening aspect. Only 4 of these 43 apps were developed in India. In a review of free apps for depression available for Android phone users in India, 278 apps were identified in the first step and information on coping with depression and stand-alone screening tools formed the 2 largest types of free apps. Features of interactive self-care apps (N=33) were reviewed further, and this exercise showed that less than 10% of the apps incorporated explicit delineation of their scope or initial screening for suitability. Slightly more than one-third of these apps included content aimed at encouraging professional help-seeking when needed or an explicit mention of their theoretical or empirical basis. Challenges for potential users were highlighted [20].

A recent survey of smartphone users in India highlighted that most of them (69%) were not aware of any mental health apps [21]. In another study involving persons with severe mental illness seeking treatment in a tertiary care setting in India and their caregivers, it was noted that health app use was low, with costs, lack of familiarity, and language being significant barriers to use [22]. An Indian study examined mental health apps in the Google Play Store between 2016 and 2020 by extracting data using various software programs. The keywords used for the search included: “mental health,” “mental illness,” “mental disorders,” “cure of mental disorder,” and “healing of mental illnesses.” As per the content analysis of the apps, the apps targeted various mental health concerns ranging from depression, anxiety, stress, posttraumatic stress, sleep, obsessive compulsive disorder, substance use, and panic symptoms to schizophrenia. This review examined basic content, number of downloads, mention of interactivity, and pricing and ratings of apps. It was observed that most app users did not leave their opinions or share their experiences, and thus, 70% of app ratings were based on a small number of users (100 or fewer users). The intervention approaches mentioned in this review ranged from relaxation, stress management, symptom tracking, calming audio, keeping a diary, interpersonal support meditation, and mood tracking. Connecting to mental health professionals as a strategy was mentioned in less than 2% of the instances. This study did not focus on examining the quality and appropriateness of content, usability, or empirical evidence-based [23].

The need for mental health app libraries independent of commercial biases has been advocated [24-26]. Lagan et al [17] systematically evaluated 278 mental health apps using the Mhealth Index and Navigation Database framework, which comprises 105 questions across 6 categories, namely, app origin or accessibility, privacy or security, inputs or outputs, clinical foundation or evidence base, features or engagements, and interoperability. The most common features offered by the 278 apps were mood tracking and journaling, with much fewer ones offering comprehensive therapeutic interventions that may be useful for addressing the diversity of user needs. Less than 25% of apps were supported by a feasibility or efficacy study [17].

From end users’ perspectives, when selecting apps, they rely mainly on ratings and reviews in app stores or on advice given through social media or word of mouth [27]. However, the lack of involvement of mental health experts in app development and insufficient attention to evidence-based development are factors that can limit the effectiveness of the apps or even pose a risk for harm due to unscientific suggestions or lack of information on limitations of app-based interventions and need for direct professional consultations.

It has been observed that many apps in commercial app stores, including low-quality ones, make it difficult for users to identify a suitable app for various mental health conditions, and evidence-based is often lacking, even for top-rated apps in app stores [28,29].

### Rationale for the Review

Studies have highlighted that most well-researched apps are not easily accessible to the public, while a bulk of apps available in app stores are not backed by research evidence or do not involve qualified mental health professionals in their conceptualization, development, or validation [30]. In contrast to the reviews on research studies on the efficacy or effectiveness of mental health apps, there have been fewer studies that have directly reviewed mental health apps themselves, which are available for end users in app stores.

Apart from the lacuna mentioned earlier, data security and privacy concerns are some of the factors that restrict the potential of this field, as mental health apps use sensitive information pertaining to personal concerns, thoughts and moods, and sleep patterns. High attrition rates and inconsistent or infrequent usage of apps are challenges noted across the globe that reduce the potential effectiveness of the apps for users, and this is particularly true of mental health apps that are completely unguided as these do not involve any human interface or assistance for use. The other challenges surrounding mental health apps in India include the diversity of languages spoken across the country and the sociocultural factors that can make it difficult to develop apps that cater to the population's diverse needs.

There is a dearth of systematic reviews of mental health apps available in app stores for Indian users. Reviews that rely on the number of downloads, user ratings, or merely app store descriptions are unlikely to be helpful in guiding the consumers of mental health apps in making informed decisions. This study is designed to address these gaps by conducting a systematic review of mental health apps accessible to Indian users, using a standardized framework for assessment. This systematic review is expected to help in the creation of a user-friendly platform to guide consumers of mental health apps in India. This is especially important as the commercial marketplace offers a confusing array of options with a plethora of apps, a diverse range of content, a lack of clarity on scope or indications for the use of specific apps, and a lack of noncommercial platforms providing detailed information that can assist users in choosing apps that serve their needs.

Hence, we aim to conduct a comprehensive and systematic review of smartphone-based mental health apps available in app stores that are accessible to Indian end users in order to (1) describe these apps in terms of characteristics such as purpose, nature of the intervention, mental health conditions focused upon, involvement of mental health professionals in development, mention of empirical basis, nudges to seek professional help as well as (2) evaluate and document the quality of these apps.

## Methods

### Ethical Considerations

The systematic review has been registered on the International Platform of Registered Systematic Review and Meta-analysis Protocols (INPLASY2024100035). An exemption from the Institute Ethics Committee (Behavioural Science Division), NIMHANS (No. NIMHANS/EC/[BEH.SC.DIV.] MEETING/2024) was sought and obtained for this review.

### App Review Approach

An approach called Target user, Evaluation focus, Connectedness and Health domain (TECH) has been developed to formulate research questions and determine eligibility criteria for systematic reviews of commercially available health apps. This is because formats such as Population, Intervention, Comparison, and Outcome (PICO) and Sample, Phenomenon of Interest, Design, Evaluation, Research (SPIDER) are appropriate primarily for a systematic review of studies examining the effectiveness of interventions and for systematic qualitative review, respectively [31]. Here TECH refers to (1) target user (the specific population in question); (2) evaluation focus (eg, app characteristics, quality, usability, techniques, or components); (3) connectedness (whether the app connects with other apps or services); and (4) health domain (eg, specific health condition or concern being focused). This approach is aimed at helping in the development of the research question and for guiding eligibility criteria for the review of apps. Applying the TECH approach in this study context, the target population broadly consists of Indian adults interested in exploring or using apps to understand or manage mental health concerns. The evaluation focus is kept intentionally broad to include descriptive information on app characteristics, content, and quality. The review intends to include stand-alone apps as well as those that may connect to services but apps that connect to wearables or other apps would fall beyond the purview of this review. The health domain focused upon in the review is broad and includes various mental health concerns and conditions, The eligibility criteria mentioned subsequently drew upon these elements of the TECH approach.

**Figure 1:**
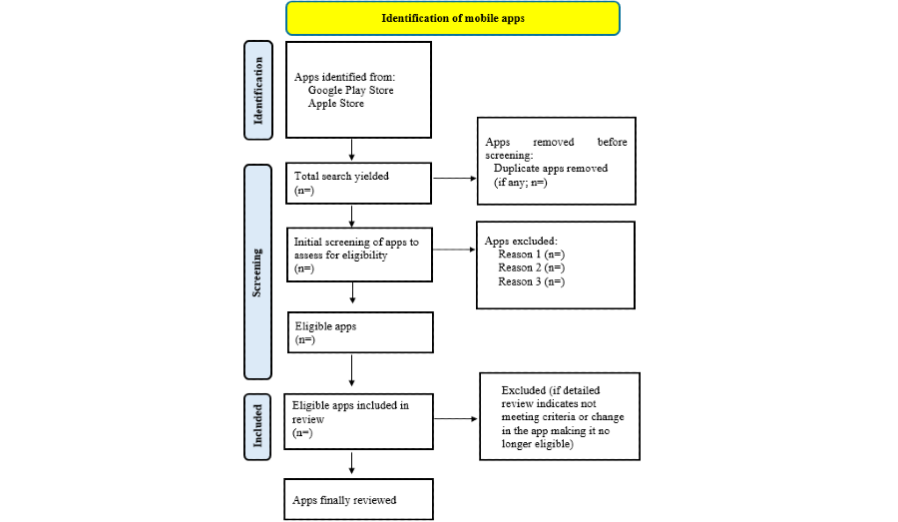
Flow diagram depicting the exclusion of apps at different stages of the review.

Since this is proposed to be a review of mental health apps and not a systematic review of research studies on mental health apps, the Protocol for App Store Systematic Reviews (PASSR) checklist will be used (see flow diagram in Figure 1) [13]. It is a combination and adaptation of the items from A Measurement Tool to Assess systematic Reviews (AMSTAR) and Preferred Reporting Items for Systematic Reviews and Meta-Analyses (PRISMA) checklists that can be applied to the systematic search of app stores for any category of apps [32,33].

### Inclusion and Exclusion Criteria

The inclusion criteria for the review of apps are as follows:

- Apps available in Google Play Store or Apple App Store for Indian adults.
- The store description of the app indicates they are offering guidance on mental health problems or therapy, information, self-help, or support and are thus relevant for the review.
- Apps available in English.
- Free apps and partially free apps (for those involving in-app purchases, what is freely available will be reviewed).

It should be noted that the number of apps that are available in any Indian language that do not have an English version will also be documented but not included in the detailed review.

The exclusion criteria for the review of apps are as follows:

- Mental health apps that are fully paid and do not have a free trial period.
- Apps available solely in a non-English language.
- Apps meant for exclusive use by health professionals (eg, for training or education).
- Apps that require any additional wearable devices or sensors.
- Apps that are solely focused on enhancing wellness without the context of mental health concerns (eg, improving time management, improving productivity, managing relationships).
- Apps available only for research participants of a given study.

The keyword-driven search was carried out during October 2024 and November 2024. Apps that are no longer available to download in January 2025 for a detailed review will be excluded.

The aforementioned search criteria have been set in order to ensure that the research objectives can be adequately addressed while also taking into account feasibility considerations for this comprehensive review. The search will be carried out on Google Play stores and Apple iOS stores as these together comprise about 99% of the Indian market share [34]. Most of the mental health apps for Indian users are in English language. Any app in an Indian language that has an English version will be included for review. India is a multilingual country with 121 languages [35]. Apps available only in a non-English language will be documented but excluded from detailed review as that would require expertise in the given language. There is a very huge and broad range of apps that are on themes related to well-being and these deserve a separate review exercise. The review is restricted to apps that focus on one or more mental health conditions to ensure a focus and enhance the feasibility of the study. Apps that require additional wearable devices are not included in this review as they can have accessibility or affordability issues. These are also likely to be vulnerable to cyberattacks and may require smart intrusion detection systems [36]. Also, apps directed at health professionals would be excluded as the purpose of the review is to evaluate apps for the public.

### App Search Strategy

The keywords used in this study are “Mental health,” “Depression,” “OCD,” “Addiction,” “Schizophrenia,” “Anxiety,” “PTSD,” “Bipolar,” “BPAD,” “CBT,” “ACT,” “DBT,” “Cognitive Behaviour Therapy,” “Acceptance and Commitment Therapy, and “Dialectical Behaviour Therapy.”

The selection of these 15 keywords was guided by the purpose of this study which was to cover a broad range of common and prevalent mental health concerns, common psychotherapy interventions as well and their commonly used acronyms. Further, a broad scan of review studies in the last 5 years on mental health apps, as well as an initial scanning of the apps in the app stores helped in finalizing the keywords.

The following search strategy will be adopted:

- Searches will be conducted in each store (marketplace) with each of the aforementioned keywords.
- Will use a clean or reset device to search the data store with a new Gmail or iOS ID.
- Details of the search dates will be recorded.
- The entire search process will be screen-recorded for reference while ensuring slow scrolling from the top to the end of the screen. To avoid missing any apps, scrolling will continue past any advertisements until all content is thoroughly recorded.
- Before the second key term search, the Play Store or Apple App Store history from settings will be cleared and reset.
- All the listed apps in the screen recordings will be carefully entered on a Microsoft Excel sheet.
- If an app is available both on the Google Play Store and Apple Stores Android and iOS phones, the version on the Google Play Store will be reviewed, and the app will be counted only once.
- Four mobile devices (2 Apple and 2 Android devices) will be used for the search.

### Initial Screening

After lists of apps on the 2 stores are generated, duplicates will be removed, and the remaining apps will be screened for eligibility using the selection criteria. This screening task will be divided among 4 members of the review team. Any doubts regarding inclusion will be resolved through joint discussions.

The apps meeting the inclusion criteria will be downloaded for detailed review. At this stage too, if any apps are found to be nonrelevant or not meeting any of the inclusion criteria will be excluded from further review.

From the final pool of apps, 6 apps will be randomly selected for joint discussion and review by a team consisting of 4 primary reviewers along with 2 clinical psychology faculty serving as mentors (with more than 10 years of overall experience and familiarity with app review processes). Following this, a new set of 6 randomly selected apps from the remaining ones will be rated independently by the primary review team and the differences in review ratings, if any, will be discussed with all the team members, including the mentors, to arrive at a consensus. This process is adopted to streamline the review process and increase interrater consistency in review and ratings through training and the use of a panel of primary reviewers and mentors. In the last step, the primary reviewers will individually review the remaining apps in the list. In addition, 2% of all the apps, (depending on the number of finally selected eligible apps) will be randomly selected and reviewed collectively by the mentor team in order to note the concordance with the ratings by the primary reviewers.

### Data Extraction

The detailed review of downloaded apps will involve documenting the following aspects in Table 1.

**Table 1:** Information of apps planned to be extracted.

Serial number	Information parameter	Coding format
1	Privacy-related: Privacy policy accessible to users Privacy terms clearly explained Mention of data-sharing policy with third parties Mention of data retention duration Mention of provision for deletion of user account	Yes or no
2	Payment requirements	Completely free to use Fully paid Involves in-app purchases
3	Nature of developer	Company Not-for-profit organizations Academic bodies Government agency Insufficient information available
4	Country of origin	Mentioned name of the country Insufficient information available
5	Release	Date of first release Insufficient information available
6	Update	Date of last update Insufficient information available
7	Downloads	Number of downloads
8	Rating	Number of reviews Average review rating
9	Log-in requirements	Yes or no
10	Language	English Non-English If in any Indian language
11	Mental health professionals’ involvement in development of the app	Clearly specified Generally mentioned Not mentioned
12	Empirical research on the app mentioned	Yes or no
13	Target users: Specified minimum age (if any) Any special group (eg, veterans)	Yes or no
14	Basic purpose of the app (as ascertained from the content and features)	Free text (eg, providing information, psychoeducation, screening, self-help, synchronous or asynchronous chat-based support, therapy or counseling support, artificial intelligence–based counseling, peer-support, symptom monitoring, tracking)
15	Mental health condition	Single mental health condition focused Multiple mental health conditions focused Not mentioned
16	Type of intervention: Type of therapy mentioned Guided or unguided	Free text (like CBT^a^, DBT^b^, and mindfulness) Yes or no
17	Components of the intervention	Single component or multiple components
18	Empirical basis of the intervention	Mentioned or not mentioned
19	Nature of the intervention Attempts to dispel common myths related to mental health and illness	Yes or no
20	Crisis management Mentioned helpline, emergency service details, or directories Basic crisis support strategies	Yes or no
21	Sociocultural appropriateness of examples used (case study)	Yes, no, or could not be ascertained
22	Inclusion of pointers on when to seek professional help	Yes or no
23	Inclusion of nudges to seek professional help when needed	Yes or no
24	Any special feature	Yes (a brief description of the feature) or no

^a^CBT: cognitive behavior therapy.

^b^DBT: dialectical behavior therapy.

Some modifications and refinements in the data extraction parameters and process may be considered if required depending on the review of the initial 15 apps by the primary reviewers.

### Evaluation of Quality of Apps Using Ratings From the Mobile Application Rating Scale

In addition to documentation on the earlier aspects, primarily related to the content, the apps will also be evaluated using the Mobile Application Rating Scale (MARS). This scale consists of 19 items across 4 domains, that is, engagement, functionality, aesthetics, and information quality [37]. Each item is rated on a 5-point Likert scale: (1) inadequate, (2) poor, (3) acceptable, (4) good, and (5) excellent. The total score and the 4 objective domains have high internal consistency. An additional 4-item subjective quality scale is also available, though it is not counted toward the total. MARS has been one of the most widely used tools across nations for the evaluation of the quality of mobile health (mHealth) apps, including mental health apps [38-40]. A recent study documented the validity of MARS by using pooled MARS data from 15 international reviews assessing the quality and content of mHealth apps in various health conditions [41].

### Strategy for Data Analysis and Synthesis

The analysis will involve the use of descriptive statistics such as frequencies, percentages, mean, and SDs. This would be in addition to the qualitative analysis of the apps as mentioned earlier through the use of MARS ratings. In addition to the overall findings, an attempt will be made to synthesize and present subgroup-wise findings based on apps with a different focus in terms of the nature of mental health conditions (eg, severe vs common mental health conditions) and those with single versus multicomponent interventions, provided there is a sufficient number of apps to form such subgroups. This study is focused on reviewing apps themselves rather than reviewing studies about apps and hence the traditional methods for assessing the risk of bias are not applicable. There is a scarcity of guidelines to assess the risk of bias in studies evaluating apps on app stores. An attempt will be made to describe the potential risk of bias at the stage of inclusion of apps for review, their detailed review by independent reviewers, and the handling of missing data while reporting findings. The risk of bias will also be documented by mentioning whether any apps being reviewed have been examined by any member of the research team for effectiveness or efficacy [13].

## Results

The search for mental health apps in virtual stores has been completed. The initial screening has been completed. The initial search yielded 5827 apps, comprising 3708 apps on the Android Play Store and 2119 apps on the Apple App Store. We expect to complete the detailed systematic review by April 2025.

## Discussion

### Significance of the Systematic Review of Mental Health Apps

In the background of a huge treatment gap, mental health apps can contribute in numerous ways to strengthen mental health care systems, empower communities with low-intensity, affordable, and accessible options for self-care for milder problems as well as enhance mental health literacy and reduce barriers to seeking mainstream mental health services; provided that the challenges related to quality, security, congruency to user needs, and user engagement are appropriately addressed [42-44]. As pointed out earlier, the current regulatory frameworks for mental health apps are still evolving and there is a plethora of apps in the commercial marketplace with little information available on their evidence or use of evidence-based content [18,20]. Hence, a review like the present one can be a stepping stone to exploring the nature of apps and subsequently coming up with suggestions and recommendations to improve the safety and quality of apps [45]. The review is not focused on outcome research on apps; instead, it delves into examining apps in terms of the nature of content, their scope, and quality. As previous research indicates, the popularity of apps and user experience do not necessarily predict sustained engagement and hence the need for systematic evaluation of apps on a comprehensive set of parameters including sociocultural appropriateness, as planned in this review [29,46,47]. It is important to highlight that the potential impact of the review is not to come up with a list of apps that are “flagged as not useful,” instead it attempts to draw the attention of the public, developers, and mental health professionals to make informed decisions for choosing, using, designing, and studying mental health apps judiciously to maximize their potential for serving the mental health needs of the population.

### Future Plans

Following the systematic review of the mental health apps in app stores, an attempt will be made to categorize them based on various parameters that may be relevant for potential end users as well as professionals who search for relevant apps on stores for specific purposes (eg, target group, main purposes, level of interactivity, functions, and scope and limits of the app). The outcome of this process will be made available to the public and professionals in a web-based portal in an easy-to-use search or filter format.

### Strengths and Limitations

This would be one of the first systematic reviews of mental health apps available in app stores for Indian users, using a comprehensive set of keywords related to mental health conditions and therapies. Also, this review would go beyond app store descriptions and indices such as user reviews and number of downloads and would entail examining the actual content, functionalities, and features of the apps through engaging a panel of raters, including mental health professionals. Unlike literature searches, searching apps in app stores have some limitations. The search can be challenging as the order in which the search results are displayed can be variable and the usual filter functions like those when reviewing research are not available. Moreover, the initial screening would be guided by the app store descriptions that may not always provide clear information being sought by the reviewers and hence may result in the exclusion of some relevant apps, although an attempt will be made to err on the side of overinclusiveness, during the initial round of screening.

### Conclusions

The review will provide a comprehensive and critical review of the smartphone apps on mental health that are available for Indian end users. The review findings are likely to have implications for end users, policy makers, developers, and researchers in the field of mental health apps.

## Abbreviations

AMSTAR: A Measurement Tool to Assess systematic Reviews
MARS: Mobile Application Rating Scale
mHealth: mobile health
PASSR: Protocol for App Store Systematic Reviews
PRISMA: Preferred Reporting Items for Systematic Reviews and Meta-Analyses
TECH: Target user, Evaluation focus, Connectedness and Health domain
